# Redetermination of MgCrO_4_·5H_2_O

**DOI:** 10.1107/S1600536813018588

**Published:** 2013-07-10

**Authors:** Matthias Weil

**Affiliations:** a Institute for Chemical Technologies and Analytics, Division of Structural Chemistry, Vienna University of Technology, Getreidemarkt 9/164-SC, A-1060 Vienna, Austria

## Abstract

The CCD-data based redetermination of the crystal structure of the title compound, magnesium chromate(VI) penta­hydrate, confirms in principle the previous study based on precession film data [Bertrand *et al.* (1971 ▶). *C. R. Hebd. Seances Acad. Sci. Serie C*, **272**, 530–533.], but with all atoms refined with anisotropic displacement parameters and with all H atoms localized. This allowed an unambiguous assignment of the hydrogen-bonding pattern. MgCrO_4_·5H_2_O adopts the MgSO_4_·5H_2_O structure type. It contains two Mg^2+^ sites on special positions with site symmetry -1, one tetra­hedral CrO_4_ group and five water mol­ecules. Four of them coordinate to the Mg^2+^ cation, and one is an uncoordinating lattice water mol­ecule. The octa­hedral environment of the Mg^2+^ cation is completed by two axial O atoms of CrO_4_ tetra­hedra. This arrangement leads to the formation of chains parallel to [011]. Adjacent chains are linked through O—H⋯O hydrogen bonds (one of them bifurcated), involving both the coordi­nating and lattice water mol­ecules, into a three-dimensional network.

## Related literature

For the original structure determination of the title compound, see: Bertrand *et al.* (1971 ▶). For hydrogen-bonding pattern in the structures of *MX*O_4_·5H_2_O compounds (*M* = Mg, Cu; *X* = S, Cr), see: Baur & Rolin (1972 ▶). For Cr—O bond length distributions in chromates(VI), see: Pressprich *et al.* (1988 ▶). For bond lengths and angles in the related structure of MgCrO_4_·11H_2_O, see: Fortes *et al.* (2013 ▶). For standardization of structure data, see: Gelato & Parthé (1987 ▶).

## Experimental

### 

#### Crystal data

MgCrO_4_·5H_2_O
*M* *_r_* = 230.39
Triclinic, 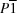

*a* = 6.1467 (3) Å
*b* = 6.3742 (4) Å
*c* = 10.7048 (6) Å
α = 75.919 (4)°
β = 81.603 (3)°
γ = 71.134 (3)°
*V* = 383.92 (4) Å^3^
*Z* = 2
Mo *K*α radiation
μ = 1.59 mm^−1^
*T* = 296 K
0.10 × 0.08 × 0.01 mm

#### Data collection

Bruker APEXII CCD diffractometer
Absorption correction: multi-scan (*SADABS*; Bruker, 2011 ▶) *T*_min_ = 0.594, *T*_max_ = 0.748
10569 measured reflections
4019 independent reflections
3340 reflections with *I* > 2σ(*I*)
*R*_int_ = 0.031

#### Refinement

*R*[*F* ^2^ > 2σ(*F* ^2^)] = 0.031
*wR*(*F* ^2^) = 0.069
*S* = 1.02
4019 reflections
143 parameters
10 restraints
H atoms treated by a mixture of independent and constrained refinement
Δρ_max_ = 1.17 e Å^−3^
Δρ_min_ = −0.72 e Å^−3^

### 

Data collection: *APEX2* (Bruker, 2011 ▶); cell refinement: *SAINT* (Bruker, 2011 ▶); data reduction: *SAINT*; program(s) used to solve structure: *SHELXS97* (Sheldrick, 2008 ▶); program(s) used to refine structure: *SHELXL97* (Sheldrick, 2008 ▶); molecular graphics: *ATOMS for Windows* (Dowty, 2008 ▶); software used to prepare material for publication: *publCIF* (Westrip, 2010 ▶).

## Supplementary Material

Crystal structure: contains datablock(s) I, global. DOI: 10.1107/S1600536813018588/br2229sup1.cif

Structure factors: contains datablock(s) I. DOI: 10.1107/S1600536813018588/br2229Isup2.hkl

Additional supplementary materials:  crystallographic information; 3D view; checkCIF report

## Figures and Tables

**Table 1:** Selected bond lengths (Å)

Mg1—O*W*3	2.0505 (9)
Mg1—O*W*2^i^	2.0656 (9)
Mg1—O4^ii^	2.0952 (9)
Mg2—O*W*5	2.0265 (10)
Mg2—O*W*1	2.0467 (8)
Mg2—O1	2.1099 (9)
Cr1—O3^iii^	1.6357 (9)
Cr1—O4^iii^	1.6554 (9)
Cr1—O1	1.6568 (9)
Cr1—O2	1.6579 (8)

Symmetry codes: (i) 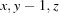; (ii) 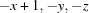; (iii) 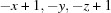.

**Table 2:** Hydrogen-bond geometry (Å, °)

*D*—H⋯*A*	*D*—H	H⋯*A*	*D*⋯*A*	*D*—H⋯*A*
O*W*1—H*W*1*A*⋯O2^iv^	0.81 (2)	1.96 (2)	2.7702 (13)	174 (2)
O*W*1—H*W*1*B*⋯O3	0.80 (2)	1.97 (2)	2.7598 (13)	169 (2)
O*W*2—H*W*2*A*⋯O1^v^	0.79 (2)	2.19 (2)	2.9014 (13)	150 (2)
O*W*2—H*W*2*A*⋯O*W*1	0.79 (2)	2.52 (2)	3.1008 (14)	132 (2)
O*W*2—H*W*2*B*⋯O*W*4	0.80 (2)	2.02 (2)	2.8173 (12)	171 (2)
O*W*3—H*W*3*A*⋯O2^iii^	0.82 (2)	1.97 (2)	2.7891 (13)	170 (2)
O*W*3—H*W*3*B*⋯O*W*4^vi^	0.81 (2)	1.99 (2)	2.7906 (14)	172 (2)
O*W*4—H*W*4*B*⋯O1^iv^	0.83 (2)	2.32 (2)	3.1205 (14)	163 (2)
O*W*4—H*W*4*A*⋯O4	0.80 (2)	2.06 (2)	2.8535 (14)	170 (2)
O*W*5—H*W*5*B*⋯O2^iii^	0.80 (2)	1.93 (2)	2.7262 (13)	173 (2)
O*W*5—H*W*5*A*⋯O3^vii^	0.79 (2)	1.96 (2)	2.7409 (12)	174 (2)

Symmetry codes: (iii) 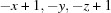; (iv) 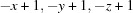; (v) 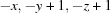; (vi) 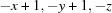; (vii) 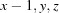.

**Fig. 1.**
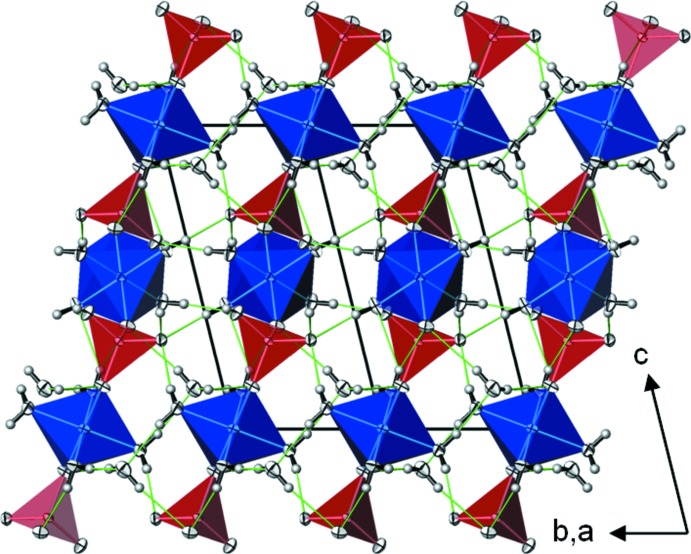
Projection of the structure of MgCrO4.5H2O along [110]. MgO6 octahedra are blue, CrO4 tetrahedra are red, O atoms are colourless. For non-H atoms, displacement parameters are given at the 90% probability level. O—H···O hydrogen bonds are indicated by green lines.

## supplementary crystallographic information

### Comment

In the current study MgCrO_4_.5H_2_O was prepared as a precursor for preparation of anhydrous MgCrO_4_. The structure of MgCrO_4_.5H_2_O has already been determined from film data (precession camera) by Bertrand *et al.* (1971), revealing isotypism with MgSO_4_.5H_2_O, CuSO_4_.5H_2_O and other *MX*O_4_.5H_2_O structures (*M* = Mg or a divalent first row transition metal; *X* = S, Se, Cr). The original refinement of MgCrO_4_.5H_2_O did not report any standard uncertainties on lattice parameters and atomic coordinates. It converged with a *R* value of 0.095, with the displacement parameters of all atoms refined isotropically and with no H-atoms localized. The geometric parameters (bond lengths, bond angles, hydrogen bonding pattern) of the crystal structures of the three isotypic salts MgSO_4_.5H_2_O, CuSO_4_.5H_2_O and MgCrO_4_.5H_2_O were compared by Baur & Rolin (1972), using the original MgCrO_4_.5H_2_O data by Bertrand *et al.* (1971) under assumption of geometrically calculated hydrogen positions for the water molecules. Therefore a redetermination of the MgCrO_4_.5H_2_O structure based on modern CCD-based intensity data seemed appropriate. The current study revealed all non-H atoms with anisotropic displacement parameters and with all H atoms localized, allowing an unambiguous assignment of the hydrogen-bonding pattern, together with more accurate bond lengths.

In a crystal chemical sense, MgCrO_4_.5H_2_O is better represented by the formula [Mg(H_2_O)_4_]CrO_4_.H_2_O. Its structure contains two Mg^2+^ cations, each located on an inversion centre, one CrO_4_^2-^ anion and five water molecules. The Mg^2+^ cations are octahedrally surrounded by four water molecules in equatorial sites and by O atoms of CrO_4_ tetrahedra in axial sites. The bridging character of the CrO_4_ tetrahedra leads to the formation of chains extending parallel to [011] (Fig. 1). The two [MgO_2_(H_2_O)_4_] octahedra are slightly distorted (Table 1), with average Mg—O bond lengths of 2.070 for Mg1 and 2.061 for Mg2. The CrO_4_ tetrahedron is likewise slightly distorted and has a mean Cr—O bond lengths of 1.658 Å, typical for chromates(VI) with isolated CrO_4_ anions (1.646 (25) Å; Pressprich *et al.* 1988). The values of bond lengths and angles of the MgO_6_ octahedron and the CrO_4_ tetrahedron in the title structure are in the same range as in the related undecahydrate MgCrO_4_.11H_2_O (Fortes *et al.*, 2013).

Neighbouring chains are linked through O—H···O hydrogen bonds, involving the coordinating water molecules as well as the lattice water molecule (OW4). The strength of most of the hydrogen bonds can be considered as medium-strong, with O···O separations between 2.7262 (13) and 2.7906 (14) Å. Somewhat weaker hydrogen bonds are also present, with O···O separations > 2.80 Å, and the longest O···O separation being 3.1205 (14) Å. It is interesting to note that HW2A protons are involved in a bifurcated hydrogen bond (Table 1).

The experimentally determined hydrogen bonding scheme of [Mg(H_2_O)_4_]CrO_4_.H_2_O is in good agreement with the one calculated and discussed previously by Baur & Rolin (1972).

### Experimental

Half-concentrated chromic acid, prepared by dissolving CrO_3_ in water, was neutralized with MgCO_3_. This solution was evaporated until dryness and the resulting solid recrystallized in water. Yellow crystals with a platy habit and edge length up to 1 mm were obtained.

### Refinement

In the original study (Bertrand *et al.*, 1971) a non-reduced setting in space group *P*1 has been used, with lattice parameters *a* = 6.384, *b* = 10.702, *c* = 6.115 Å, *α* = 81.55, *β* = 108.75, *γ* = 104.333 °. For the present study the unit-cell parameters were transformed into the reduced cell using the transformation matrix (0 0 1, 1 0 0, 0 1 0). For refinement, the atomic coordinates of the original determination were used as starting parameters. They were finally standardized with *STRUCTURE TIDY* (Gelato & Parthé, 1987). All H atoms were discernible from difference maps. Their coordinates were refined with distance restraints of *d*(O—H) = 0.82 (5) Å, with individual *U_iso_* parameters for each H atom. One reflection (0 0 1) was affected by the beam stop and was omitted from the refinement.

### Crystal data

**Table:**

MgCrO_4_·5H_2_O	*Z* = 2
*M**_r_* = 230.39	*F*(000) = 236
Triclinic, *P*1	*D*_x_ = 1.993 Mg m^−^^3^
Hall symbol: -P 1	Mo *K*α radiation, λ = 0.71073 Å
*a* = 6.1467 (3) Å	Cell parameters from 3623 reflections
*b* = 6.3742 (4) Å	θ = 3.5–39.7°
*c* = 10.7048 (6) Å	µ = 1.59 mm^−^^1^
α = 75.919 (4)°	*T* = 296 K
β = 81.603 (3)°	Plate, yellow
γ = 71.134 (3)°	0.10 × 0.08 × 0.01 mm
*V* = 383.92 (4) Å^3^	

### Data collection

**Table:**

Bruker APEXII CCD diffractometer	4019 independent reflections
Radiation source: fine-focus sealed tube	3340 reflections with *I* > 2σ(*I*)
Graphite monochromator	*R*_int_ = 0.031
ω– and φ–scans	θ_max_ = 37.5°, θ_min_ = 3.5°
Absorption correction: multi-scan (*SADABS*; Bruker, 2011)	*h* = −10→10
*T*_min_ = 0.594, *T*_max_ = 0.748	*k* = −10→10
10569 measured reflections	*l* = −17→18

### Refinement

**Table:**

Refinement on *F*^2^	Primary atom site location: isomorphous structure methods
Least-squares matrix: full	Hydrogen site location: difference Fourier map
*R*[*F*^2^ > 2σ(*F*^2^)] = 0.031	H atoms treated by a mixture of independent and constrained refinement
*wR*(*F*^2^) = 0.069	*w* = 1/[σ^2^(*F*_o_^2^) + (0.0263*P*)^2^ + 0.1663*P*] where *P* = (*F*_o_^2^ + 2*F*_c_^2^)/3
*S* = 1.02	(Δ/σ)_max_ < 0.001
4019 reflections	Δρ_max_ = 1.17 e Å^−^^3^
143 parameters	Δρ_min_ = −0.72 e Å^−^^3^
10 restraints	

### Special details

**Table:**

Geometry. All e.s.d.'s (except the e.s.d. in the dihedral angle between two l.s. planes) are estimated using the full covariance matrix. The cell e.s.d.'s are taken into account individually in the estimation of e.s.d.'s in distances, angles and torsion angles; correlations between e.s.d.'s in cell parameters are only used when they are defined by crystal symmetry. An approximate (isotropic) treatment of cell e.s.d.'s is used for estimating e.s.d.'s involving l.s. planes.
Refinement. Refinement of *F*^2^ against ALL reflections. The weighted *R*-factor *wR* and goodness of fit *S* are based on *F*^2^, conventional *R*-factors *R* are based on *F*, with *F* set to zero for negative *F*^2^. The threshold expression of *F*^2^ > σ(*F*^2^) is used only for calculating *R*-factors(gt) *etc*. and is not relevant to the choice of reflections for refinement. *R*-factors based on *F*^2^ are statistically about twice as large as those based on *F*, and *R*- factors based on ALL data will be even larger.

### Fractional atomic coordinates and isotropic or equivalent isotropic displacement parameters (Å^2^)

**Table:**

	*x*	*y*	*z*	*U*_iso_*/*U*_eq_	
Mg1	0.0000	0.0000	0.0000	0.00672 (11)	
Mg2	0.0000	0.5000	0.5000	0.00587 (10)	
Cr1	0.35414 (3)	0.02843 (3)	0.709355 (18)	0.00508 (4)	
O1	0.17472 (14)	0.28496 (15)	0.66131 (9)	0.00919 (15)	
O2	0.61762 (13)	0.04795 (15)	0.70554 (9)	0.00825 (15)	
O3	0.64389 (14)	0.14163 (16)	0.38351 (9)	0.00978 (16)	
O4	0.72867 (14)	0.07678 (16)	0.14081 (9)	0.00919 (15)	
OW1	0.30276 (14)	0.54057 (17)	0.40541 (10)	0.01038 (16)	
HW1A	0.331 (4)	0.660 (3)	0.378 (2)	0.029 (6)*	
HW1B	0.413 (3)	0.435 (3)	0.397 (2)	0.032 (6)*	
OW2	0.16925 (14)	0.70426 (17)	0.12175 (9)	0.01025 (16)	
HW2A	0.118 (4)	0.675 (4)	0.1938 (16)	0.027 (6)*	
HW2B	0.305 (3)	0.669 (4)	0.130 (2)	0.026 (5)*	
OW3	0.16025 (16)	0.18082 (17)	0.06898 (10)	0.01161 (17)	
HW3A	0.240 (3)	0.115 (3)	0.1307 (17)	0.024 (5)*	
HW3B	0.221 (4)	0.263 (4)	0.016 (2)	0.038 (7)*	
OW4	0.65171 (16)	0.54576 (17)	0.13300 (10)	0.01237 (17)	
HW4A	0.689 (4)	0.414 (3)	0.130 (2)	0.036 (7)*	
HW4B	0.714 (4)	0.560 (4)	0.1924 (18)	0.030 (6)*	
OW5	0.03331 (14)	0.23596 (17)	0.41734 (10)	0.01081 (16)	
HW5A	−0.076 (3)	0.201 (4)	0.413 (2)	0.023 (5)*	
HW5B	0.143 (3)	0.156 (3)	0.384 (2)	0.029 (6)*	

### Atomic displacement parameters (Å^2^)

**Table:**

	*U*^11^	*U*^22^	*U*^33^	*U*^12^	*U*^13^	*U*^23^
Mg1	0.0071 (2)	0.0069 (3)	0.0059 (3)	−0.00218 (18)	−0.00012 (17)	−0.0010 (2)
Mg2	0.0065 (2)	0.0051 (3)	0.0058 (3)	−0.00195 (18)	−0.00013 (17)	−0.0007 (2)
Cr1	0.00505 (6)	0.00478 (8)	0.00506 (8)	−0.00132 (5)	−0.00015 (5)	−0.00072 (6)
O1	0.0101 (3)	0.0067 (4)	0.0085 (4)	−0.0003 (3)	−0.0012 (3)	−0.0001 (3)
O2	0.0072 (3)	0.0092 (4)	0.0093 (4)	−0.0038 (3)	−0.0002 (3)	−0.0021 (3)
O3	0.0102 (3)	0.0092 (4)	0.0113 (4)	−0.0021 (3)	−0.0015 (3)	−0.0054 (3)
O4	0.0100 (3)	0.0084 (4)	0.0071 (4)	−0.0023 (3)	0.0017 (3)	0.0004 (3)
OW1	0.0078 (3)	0.0071 (4)	0.0147 (4)	−0.0024 (3)	0.0022 (3)	−0.0010 (3)
OW2	0.0094 (3)	0.0116 (4)	0.0074 (4)	−0.0020 (3)	−0.0003 (3)	0.0006 (3)
OW3	0.0143 (4)	0.0125 (4)	0.0099 (4)	−0.0075 (3)	−0.0038 (3)	0.0007 (3)
OW4	0.0140 (4)	0.0081 (4)	0.0141 (5)	−0.0029 (3)	0.0001 (3)	−0.0018 (3)
OW5	0.0078 (3)	0.0105 (4)	0.0162 (5)	−0.0024 (3)	0.0002 (3)	−0.0075 (3)

### Geometric parameters (Å, º)

**Table:**

Mg1—OW3^i^	2.0505 (9)	Mg2—OW1	2.0467 (8)
Mg1—OW3	2.0505 (9)	Mg2—OW1^vi^	2.0467 (8)
Mg1—OW2^ii^	2.0656 (9)	Mg2—O1	2.1099 (9)
Mg1—OW2^iii^	2.0656 (9)	Mg2—O1^vi^	2.1099 (9)
Mg1—O4^iv^	2.0952 (9)	Cr1—O3^vii^	1.6357 (9)
Mg1—O4^v^	2.0952 (9)	Cr1—O4^vii^	1.6554 (9)
Mg2—OW5	2.0265 (10)	Cr1—O1	1.6568 (9)
Mg2—OW5^vi^	2.0265 (10)	Cr1—O2	1.6579 (8)
			
OW3^i^—Mg1—OW3	180.00 (5)	OW5^vi^—Mg2—O1^vi^	92.35 (4)
OW3^i^—Mg1—OW2^ii^	90.97 (4)	OW1—Mg2—O1^vi^	88.90 (4)
OW3—Mg1—OW2^ii^	89.03 (4)	OW1^vi^—Mg2—O1^vi^	91.10 (4)
OW3^i^—Mg1—OW2^iii^	89.03 (4)	O1—Mg2—O1^vi^	180.00 (3)
OW3—Mg1—OW2^iii^	90.97 (4)	O3^vii^—Cr1—O4^vii^	109.01 (5)
OW2^ii^—Mg1—OW2^iii^	180.00 (7)	O3^vii^—Cr1—O1	111.25 (5)
OW3^i^—Mg1—O4^iv^	91.55 (4)	O4^vii^—Cr1—O1	108.90 (5)
OW3—Mg1—O4^iv^	88.45 (4)	O3^vii^—Cr1—O2	109.30 (4)
OW2^ii^—Mg1—O4^iv^	88.19 (4)	O4^vii^—Cr1—O2	109.31 (4)
OW2^iii^—Mg1—O4^iv^	91.81 (4)	O1—Cr1—O2	109.05 (4)
OW3^i^—Mg1—O4^v^	88.45 (4)	Cr1—O1—Mg2	142.08 (6)
OW3—Mg1—O4^v^	91.55 (4)	Cr1^vii^—O4—Mg1^viii^	140.12 (5)
OW2^ii^—Mg1—O4^v^	91.81 (4)	Mg2—OW1—HW1A	126.4 (16)
OW2^iii^—Mg1—O4^v^	88.19 (4)	Mg2—OW1—HW1B	121.9 (17)
O4^iv^—Mg1—O4^v^	180.00 (5)	HW1A—OW1—HW1B	111 (2)
OW5—Mg2—OW5^vi^	180.00 (5)	Mg1^ix^—OW2—HW2A	119.3 (16)
OW5—Mg2—OW1	90.91 (4)	Mg1^ix^—OW2—HW2B	122.2 (15)
OW5^vi^—Mg2—OW1	89.09 (4)	HW2A—OW2—HW2B	102 (2)
OW5—Mg2—OW1^vi^	89.09 (4)	Mg1—OW3—HW3A	118.2 (15)
OW5^vi^—Mg2—OW1^vi^	90.91 (4)	Mg1—OW3—HW3B	117.0 (18)
OW1—Mg2—OW1^vi^	180.0	HW3A—OW3—HW3B	111 (2)
OW5—Mg2—O1	92.35 (4)	HW4A—OW4—HW4B	109 (2)
OW5^vi^—Mg2—O1	87.65 (4)	Mg2—OW5—HW5A	119.5 (16)
OW1—Mg2—O1	91.10 (4)	Mg2—OW5—HW5B	131.6 (16)
OW1^vi^—Mg2—O1	88.90 (4)	HW5A—OW5—HW5B	109 (2)
OW5—Mg2—O1^vi^	87.65 (4)		

Symmetry codes: (i) −*x*, −*y*, −*z*; (ii) −*x*, −*y*+1, −*z*; (iii) *x*, *y*−1, *z*; (iv) *x*−1, *y*, *z*; (v) −*x*+1, −*y*, −*z*; (vi) −*x*, −*y*+1, −*z*+1; (vii) −*x*+1, −*y*, −*z*+1; (viii) *x*+1, *y*, *z*; (ix) *x*, *y*+1, *z*.

### Hydrogen-bond geometry (Å, º)

**Table:**

*D*—H···*A*	*D*—H	H···*A*	*D*···*A*	*D*—H···*A*
O*W*1—H*W*1*A*···O2^x^	0.81 (2)	1.96 (2)	2.7702 (13)	174 (2)
O*W*1—H*W*1*B*···O3	0.80 (2)	1.97 (2)	2.7598 (13)	169 (2)
O*W*2—H*W*2*A*···O1^vi^	0.79 (2)	2.19 (2)	2.9014 (13)	150 (2)
O*W*2—H*W*2*A*···O*W*1	0.79 (2)	2.52 (2)	3.1008 (14)	132 (2)
O*W*2—H*W*2*B*···O*W*4	0.80 (2)	2.02 (2)	2.8173 (12)	171 (2)
O*W*3—H*W*3*A*···O2^vii^	0.82 (2)	1.97 (2)	2.7891 (13)	170 (2)
O*W*3—H*W*3*B*···O*W*4^xi^	0.81 (2)	1.99 (2)	2.7906 (14)	172 (2)
O*W*4—H*W*4*B*···O1^x^	0.83 (2)	2.32 (2)	3.1205 (14)	163 (2)
O*W*4—H*W*4*A*···O4	0.80 (2)	2.06 (2)	2.8535 (14)	170 (2)
O*W*5—H*W*5*B*···O2^vii^	0.80 (2)	1.93 (2)	2.7262 (13)	173 (2)
O*W*5—H*W*5*A*···O3^iv^	0.79 (2)	1.96 (2)	2.7409 (12)	174 (2)

Symmetry codes: (iv) *x*−1, *y*, *z*; (vi) −*x*, −*y*+1, −*z*+1; (vii) −*x*+1, −*y*, −*z*+1; (x) −*x*+1, −*y*+1, −*z*+1; (xi) −*x*+1, −*y*+1, −*z*.
